# Age-specific associations between environmental factors and epistaxis

**DOI:** 10.3389/fpubh.2022.966461

**Published:** 2022-10-19

**Authors:** Eun-Jin Ahn, Hyun Jin Min

**Affiliations:** ^1^Department of Anesthesiology and Pain Medicine, Chung-Ang University Hospital, Chung-Ang University College of Medicine, Seoul, South Korea; ^2^Department of Otorhinolaryngology-Head and Neck Surgery, Chung-Ang University Hospital, Chung-Ang University College of Medicine, Seoul, South Korea

**Keywords:** age group, air pollutants, environmental factors, epistaxis, meteorological factors

## Abstract

**Objective:**

Several studies have demonstrated that environmental factors, such as meteorological factors and air pollutants, are closely associated with epistaxis. However, age-specific associations between environmental factors and epistaxis have not yet been evaluated. This study aimed to evaluate the associations between individual meteorological factors and air pollutants and epistaxis, by age.

**Study design:**

A retrospective cohort study.

**Setting:**

Records of patients covered by the Korean National Health Insurance Service who visited our hospital for epistaxis between January 1, 2002, and December 31, 2015, were retrospectively reviewed.

**Methods:**

The 46,628 enrolled patients were divided into four age groups: age group 0 (<18 years, *N* = 19,580); age group 1 (18–40 years, *N* = 10,978); age group 2 (41–70 years, *N* = 13,395); and age group 3 (>70 years, *N* = 2,675). Cases of epistaxis and data on environmental factors were analyzed according to the day, month, and year. Stepwise logistic regression was performed to identify the environmental risk factors for epistaxis in each age group.

**Results:**

Age group 0 had the highest number of patients with epistaxis, whereas age group 3 had the lowest. Relative humidity, temperature, concentrations of particulate matter (PM10) and sulfur dioxide, sunshine duration, and wind speed were significantly associated with the occurrence of epistaxis in the study population. However, analysis according to age group showed that the meteorological factors and air pollutants associated with epistaxis were different in each age group.

**Conclusion:**

We suggest that the environmental risk factors for epistaxis should be differentially analyzed according to age.

## Introduction

Epistaxis is a frequent complaint and the leading cause of visits to the otorhinolaryngology department (1, 2). Multiple factors, such as infection, allergy, trauma, use of anticoagulants, and hypertension, have been evaluated as risk factors for epistaxis (3, 4). It has been suggested that environmental factors, such as meteorological factors and air pollutants, are important factors that are closely associated with the occurrence of epistaxis and hospital visits due to epistaxis (5, 6). Nunez et al. demonstrated that the monthly rate of admissions for epistaxis was closely associated with the monthly ambient temperature (7). In addition, Reddy et al. reported that temperature and water vapor pressure were significantly associated with the rate of admission owing to epistaxis (8). The incidence of epistaxis is also associated with air pollutants, such as particulate matter PM2.5 (≤ 2.5 μm) and PM10 (≤ 10 μm) (9). However, the results of previous studies on the effects of environmental factors on the incidence of epistaxis are inconsistent. For example, Yu et al. reported that all air temperature values showed a significantly strong positive correlation with the occurrence of epistaxis (10), whereas a previous study indicated that temperature is inversely associated with the rate of hospital visitation for epistaxis (11). Another study showed no correlation between the ambient temperature and the rate of hospital visitation for epistaxis (12). Bray et al. reported a positive relationship between PM10 and ozone (O3) levels and epistaxis (13). However, Akdogan et al. reported a negative correlation between the rate of hospital visitation for epistaxis and the PM10 and sulfur dioxide (SO2) levels (12). One reason for these disparate findings may be the heterogeneity in the age groups of the participants included in these studies.

Epistaxis has a bimodal peak incidence. Additionally, the mechanisms of epistaxis and its risk factors could vary with age (10). To evaluate the differential effects of each meteorological factor and air pollutant on epistaxis according to age, large population-based multicenter medical data are essential. Thus, this study aimed to evaluate the association between individual meteorological factors, air pollutants, and the occurrence of epistaxis in different age groups using a National Health Insurance Service (NHIS)-based cohort.

## Materials and methods

### Ethical considerations

This study was approved by the Institutional Review Board of Chung-Ang University Hospital (2005-015-19314) and the review board waived the requirement for informed consent. The South Korean NHIS approved the use of its database for this study (NHIS-2020-2-178).

### Data source

The study population was selected from the NHIS national sample cohort (NHIS-NSC), an administrative nationwide cohort established by the National Health Informational Database (NHID) in South Korea. The NHIS is a single health insurer covering ~97% of all Koreans and is managed by the Korean government. NHID, created by NHIS, is a public database of the entire South Korean population. It contains information and records pertaining to health care utilization, health screening, sociodemographic, and mortality data between 2002 and 2015. NHID was first established using the data of approximately one million national health insurance subscribers and medical aid beneficiaries in the system as of 2002, as well as their follow-up data from 2002 to 2013. The second version of NHIS-NSC, which has been available since 2017, comprises 14 years of follow-up data, including 2015.

### Inclusion and exclusion criteria

Patients with a history of epistaxis (International Classification of Diseases [ICD]-10 code R04.0) were initially reviewed in this study (*N* = 82,969). Patients with the following conditions were excluded: (1) possible posterior epistaxis defined as ICD-10 code R04.0 with the procedural code for general anesthesia (L1211 or L1212) (*N* = 963); and (2) possible causes of secondary epistaxis, including bleeding disorders, presence of a foreign body in the nose, history of trauma, tumors, cardiovascular system diseases, or other systemic diseases (ICD-10 codes C860, C833, C902, C923, C966, C722, C433, S00, S01, S02, C30, C31, C65, C66, C67, C68, D65, D66, D67, D68, S00, S01, S02, S05) (*N* = 35,378). We only included patients who had records of their domicile in Seoul, from where we obtained meteorological, and air pollution data to avoid any undue influence from meteorological and air pollution differences between districts.

### Exposure variables

We obtained and analyzed data on the following patient characteristics: sex, age, domicile, and date of the hospital visit. Meteorological and air pollution data were obtained from the Korea Meteorological Administration (https://web.kma.go.kr/eng/index.jsp). Data on the daily concentrations of PM10 (μg/m^3^), carbon monoxide (CO) (ppm), O3 (ppm), SO2 (ppm), and nitrogen dioxide (NO2) (ppm), and the average ground temperature (°C), lowest temperature (°C), highest temperature (°C), highest wind speed (hhmi), average wind speed (m/s), sunshine duration (h), average relative humidity (%), average air pressure (hPa), average temperature (°C), and maximum wind speed (m/s) were analyzed for the period between January 1, 2002, and December 31, 2015. Concurrent presentation of sinonasal diseases, such as chronic sinusitis (ICD-10 code J32), acute sinusitis (ICD-10 code J01), rhinitis (ICD-10 code J31), and septal deviation (ICD-10 code J342), was also reviewed.

### Assessment of outcomes

The primary outcome was the daily number of epistaxis cases. The number of epistaxis cases was defined as the number of first hospital visits for epistaxis. The epidemiology of the outcome was also analyzed on a monthly as well as an annual basis.

### Statistical analysis

All statistical analyses were conducted using SAS 9.3 (SAS Institute, Cary, NC, USA). Continuous variables are presented as median (range), and comparisons between groups were performed using the Mann–Whitney *U* test. Descriptive variables were analyzed using the chi-square test. Stepwise linear regression models were constructed using the occurrence of epistaxis as the dependent variable to identify the meteorological factors associated with epistaxis. At each step, variables were chosen based on P-values, and a P-value threshold of 0.05 was used to set a limit on the total number of variables included in the final model. All statistical tests were two-sided and statistical significance was set at 0.05. When performing stepwise regression, we only included patients who had a record of their domicile in Seoul.

## Results

**Figure 1:**
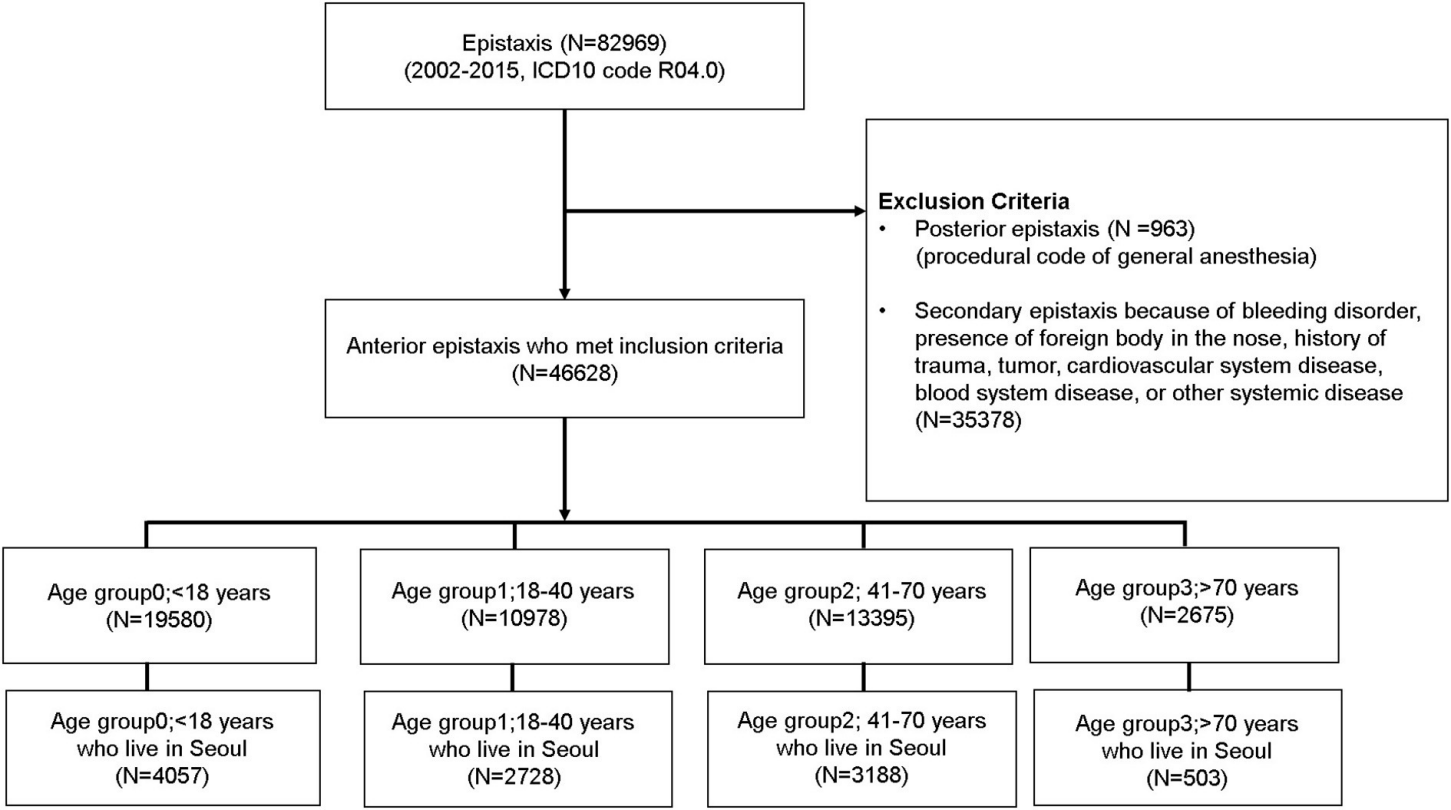
Cohort selection and study design. Patients with epistaxis who visited the hospital were selected from the National Health Informational Database and were divided into four groups according to age.

The selection of the study cohort is summarized in Figure 1. In total, 82,969 cases with epistaxis as the primary diagnosis were listed in the NHIS-NSC database. We excluded 963 cases of massive posterior bleeding that needed surgery under general anesthesia and 35,378 cases of possible secondary epistaxis. Thus, a total of 46,628 eligible cases of epistaxis were included in the study. These cases were divided into four groups according to age: age group 0 (<18 years, *N* = 19,580); age group 1 (18–40 years, *N* = 10,978); age group 2 (41–70 years, *N* = 13,395); and age group 3 (> 70 years, *N* = 2,675). Among the 46,628 eligible patients with epistaxis, only 10,476 who had a domicile in Seoul were included in the study. The final number of cases that were chosen for the study and their group-wise distribution was as follows: age group 0 (<18 years, *N* = 4,057); age group 1 (18–40 years, *N* = 2,728); age group 2 (41–70 years, *N* = 3,188); and age group 3 (> 70 years, *N* = 503) (Figure 1). Children were covered by age group 0. All adults, except geriatric cases, were divided into two groups (age groups 1 and 2). Considering that the prevalence and impact of hypertension on daily life have been reported to be particularly increased in individuals older than 70 years (14), we defined geriatrics (age group 3) as individuals older than 70 years. In this group, the occurrence of epistaxis may be strongly influenced by underlying undetected hypertension when compared with the other age groups (14, 15).

**Figure 2:**
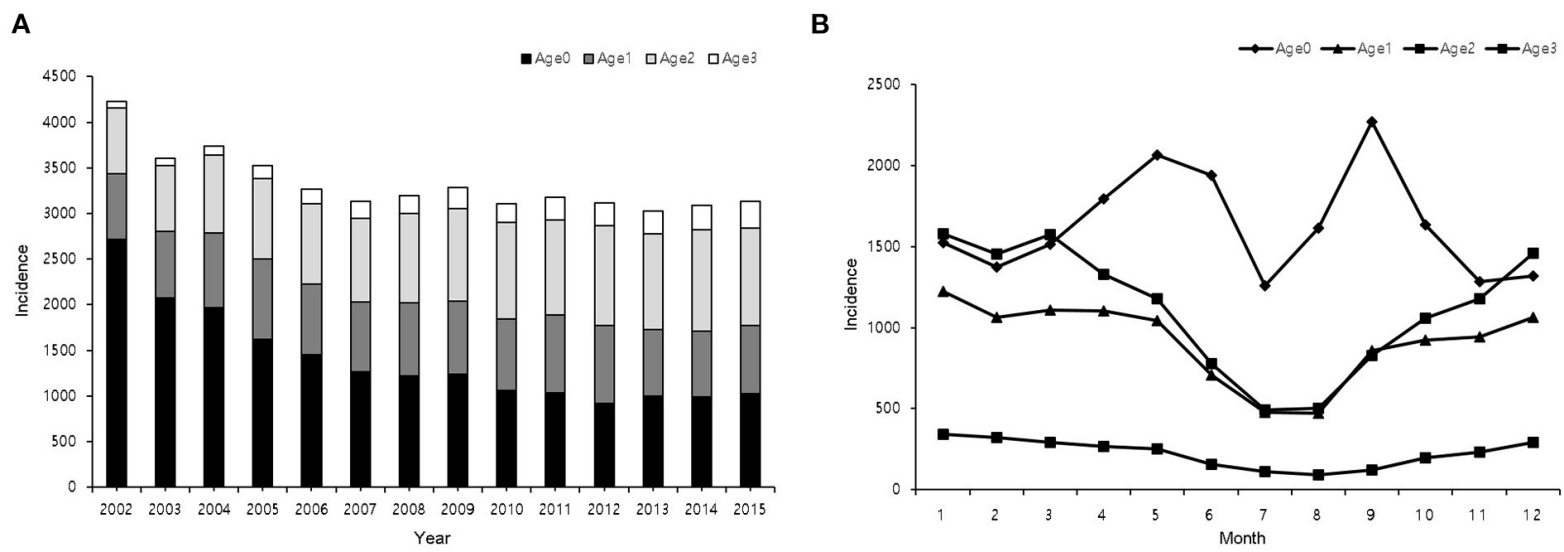
Age distribution of patients with epistaxis who visited the hospital from 2002 to 2015. **(A)** Annual distribution of patients according to age. **(B)** Monthly distribution of patients according to age.

The annual and monthly numbers of epistaxis cases according to the age groups are shown in Figure 2. The mean annual number of epistaxis cases was 3,330.57 ± 335 (Figure 2A). Analysis of the number of epistaxis cases according to the age groups showed that the annual incidence of epistaxis was different between different age groups. It was the highest in age group 0 (*N* = 19,580, 41.99%) and lowest in age group 3 (*N* = 2,675, 5.74%) (Figure 2A). The mean monthly number of epistaxis cases was 3885.67 ± 737 (Figure 2B). The number of epistaxis cases was the highest in January and lowest in July. In age group 0, the number of epistaxis cases was the highest in September (*N* = 2,270, 11.6%), followed by May (*N* = 2,065, 10.6%), and lowest in July (*N* = 1,256, 6.4%). In age group 1, the number of epistaxis cases was the highest in January (*N* = 1,223, 11.1%) and lowest in July (*N* = 476, 4.3%) and August (*N* = 471, 4.3%). In age group 2 (41–70 years old), the number of epistaxis cases was the highest in January (*N* = 1,576, 11.8%) and lowest in August (*N* = 504, 3.8%) and July (*N* = 494, 3.7%). In age group 3, the number of epistaxis cases was the highest in January (*N* = 342, 12.8%) and lowest in July (*N* = 110, 4.1%), and August (*N* = 92, 3.4%).

The mean monthly values of meteorological factors and air pollutants are described in Supplementary Table 1. The mean temperature was the highest in August, which is in the summer season in Korea, and lowest in January (winter). Wind speed was the highest in April (spring), whereas the sunshine duration was the highest in May (spring). Average relative humidity was the highest in July (summer) and lowest in February (winter). Atmospheric pressure was the highest in January and lowest in July. Regarding air pollutants, the levels of PM10 and NO2 were the highest in April, those of CO and SO2 were the highest in January, and that of O3 was the highest in June (Supplementary Table 1).

Stepwise regression analysis showed that average relative humidity, both the lowest and the average air temperature, PM10 and SO2 concentrations, sunshine duration, and average wind speed were significantly associated with epistaxis (*P* < 0.05) (Table 1). Analysis of the effect of each variable on epistaxis according to age showed that average wind speed, average relative humidity, levels of PM10, sunshine duration, SO2 concentration, average ground temperature, and lowest temperature were significantly associated with epistaxis in age group 0 (*P* < 0.05) (Table 2). In age group 1, the lowest temperature, average relative humidity, concentration of PM10, mean temperature, sunshine duration, and SO2 concentration were significantly associated with epistaxis (*P* < 0.05). In age group 2, the lowest and highest temperature, maximum wind speed, and average atmospheric pressure were significantly associated with epistaxis (*P* < 0.05). In age group 3, the lowest temperature, maximum wind speed, and highest temperature were related to epistaxis (*P* < 0.05).

**Table 1:** Stepwise regression model for the environmental risk factors for epistaxis in the study population.

**Sl. No**	**Label**	***F***	**Pr > F**
1	Average relative humidity (%)	274.47	<0.0001
2	Lowest air temperature (°C)	59.01	<0.0001
3	PM_10_ (μg/m^3^)	25.61	<0.0001
4	Average air temperature (°C)	20.21	<0.0001
5	Sunshine duration (h)	34.18	<0.0001
6	SO_2_ (ppm)	11.47	0.0007
7	Average wind speed (m/s)	4.88	0.027
8	Average ground temperature (°C)	2.73	0.098

PM_10_, particulate matter with diameter ≤ 10 μm; SO_2_, sulfur dioxide.

**Table 2:** Stepwise regression model for the environmental risk factors of epistaxis in each age group.

	**Step**	**Label**	***F***	**Pr > F**
Age group 0 (<18 years of age)	1	Average wind speed (m/s)	73.84	<0.0001
	2	Average relative humidity (%)	87.86	<0.0001
	3	PM_10_ (μg/m^3^)	51.09	<0.0001
	4	Sunshine duration (h)	43.44	<0.0001
	5	SO_2_ (ppm)	28.14	<0.0001
	6	Average ground temperature (°C)	9.75	0.0018
	7	Lowest temperature (°C)	7.73	0.0055
	8	Mean temperature (°C)	3.37	0.067
Age group 1 (18–40 years of age)	1	Lowest temperature (°C)	339.44	<0.0001
	2	Average relative humidity (%)	59.61	<0.0001
	3	PM_10_ (μg/m^3^)	10.12	0.0015
	4	Mean temperature (°C)	6.31	0.012
	5	Sunshine duration (h)	7.7	0.0055
	6	SO_2_ (ppm)	4.04	0.044
Age group 2 (41–70 years of age)	1	Lowest temperature (°C)	799.8	<0.0001
	2	Highest temperature (°C)	40.67	<0.0001
	3	Maximum wind speed (m/s)	7.77	0.0053
	4	Average atmospheric pressure (hPa)	10.63	0.0011
	5	Average relative humidity (%)	3.81	0.051
	6	Sunshine duration (h)	6.5	0.011
Age group 3 (> 70 years of age)	1	Lowest temperature (°C)	263.22	<0.0001
	2	Maximum wind speed (m/s)	34	<0.0001
	3	Highest temperature (°C)	10.94	0.0009

PM_10_, particulate matter with diameter ≤ 10 μm; SO_2_, sulfur dioxide.

## Discussion

In this study, we evaluated the prevalence of epistaxis and its association with meteorological factors and air pollutants in different age groups. In general, we found that the number of epistaxis cases was higher in younger individuals than in older ones and that the relationship between meteorological factors and air pollutants and epistaxis differed according to age. Additionally, we found that the monthly pattern of the incidence of epistaxis in the older population differed from that of children and adults, suggesting that seasonal variations have different effects on the incidence of epistaxis in each age group. The main strength of this study is that we excluded cases of secondary epistaxis and minimized the effect of hypertension. We enrolled primary epistaxis cases only to ensure direct evaluation of the association between meteorological factors and air pollutants, and epistaxis.

In a previous study, Mohamad et al. reported that emergency department visits for epistaxis increase with age (16). Patients older than 65 years are more likely to present with epistaxis in the emergency department. This is contradictory to the results of the present study, which indicated that the number of hospital visits for epistaxis was the lowest in patients older than 70 years (age group 3). This difference may be attributable to the exclusion of cases of posterior bleeding in the present study, which may be closely associated with hypertension in the old age group, and the inclusion of such cases in the study by Mohamad et al. We surmise that if the effect of hypertension is excluded, the prevalence of epistaxis may be higher in younger people than in people older than 70 years.

Regarding meteorological factors, average relative humidity, temperature factors (lowest, average air temperature), the concentration of PM10, the duration of sunshine, the levels of SO2, and average wind speed (*P* < 0.05) were significantly associated with epistaxis in the study population. We found that average relative humidity, lowest air temperature, sunshine duration, concentration of SO_2_ and average wind speed negatively correlated with the epistaxis, and concentration of PM10, average air temperature, and average ground temperature positively correlated with the epistaxis (data not shown). Similarly, a previous study performed in Korea indicated that temperature, wind speed, and relative humidity were associated with epistaxis; however, age was not considered in that study (6). In the present study, the association between each meteorological factor and epistaxis was different according to age group. In age group 0, the pediatric age group (0–18 years), the average wind speed was the most significant factor associated with epistaxis. In adult groups (age groups 1, 2, and 3; older than 20 years), the lowest temperature was the most important factor associated with epistaxis. Furthermore, several meteorological factors, including wind speed, humidity, sunshine duration, and air and temperature factors, were significantly associated with epistaxis in the pediatric age group (age group 0) compared to other age groups. Unlike age group 0, only temperature and wind speed were significantly associated with epistaxis in age group 3. These findings may suggest that as younger people may spend more time outdoors than older people, they are more exposed to various meteorological factors. In their study of the relationship between meteorological factors and epistaxis in children and adults, Yu et al. reported that temperature is inversely and positively associated with the rate of hospital visitation for epistaxis in pediatric and adult patients, respectively (10).

A wide range of chemicals, particulate matter, and gaseous air pollutants are present in the atmosphere and may pose a significant health risk for the human population (17). As the nasal passage is the first part of the respiratory tract that makes contact with the environment, the nasal epithelium may be damaged by airborne environmental pollutants, possibly leading to epistaxis (17, 18). In the present study, we evaluated the concentrations of O3, SO2, NO2, CO, and PM10 as potential factors associated with the occurrence of epistaxis because they are regularly monitored in Korea. We found that only the concentrations of PM10 and SO2 were associated with epistaxis in the study population. However, the association between the concentrations of PM10 and SO2 and epistaxis was statistically significant in age groups 0 and 1 (children and young adults; age < 39 years) but not in older age groups. In a previous study conducted on pediatric patients, the incidence of epistaxis was negatively correlated with PM2.5, PM10, SO2, NO2, and CO, which was positively correlated with O3 (*P* < 0.05) (9). In that study, the concentrations of air pollutants showed a downward trend, lower in summer than in the other three seasons, except for the concentration of O3, which showed an upward trend in summer (9). In the present study, the concentration of NO2 was higher in the spring and autumn seasons, whereas the concentrations of O3 and PM10 were higher in the spring season, in which an increased incidence of epistaxis was recorded in the younger population (Figure 2B). However, the specific concentrations of the air pollutants reported in previous studies differ from those in the present study. We suggest that the concentrations of air pollutants, climate variables, and the age of the population should be considered together in the evaluation of the effects of air pollutants on the incidence of epistaxis.

Several studies have proven that allergic rhinitis is closely associated with epistaxis (19, 20). Other sinonasal diseases could also influence the occurrence of epistaxis. For example, the association of chronic sinusitis with an increased incidence of epistaxis has been reported (21). When we evaluated the relationship between epistaxis and concurrent sinonasal diseases such as chronic sinusitis, acute sinusitis, chronic rhinitis, and septal deviation, we did not observe any significant association between these sinonasal comorbidities and epistaxis in any of the age groups (Supplementary Table 2). The relationship between sinonasal diseases (other than allergic rhinitis) and epistaxis could be another interesting research topic for future studies.

Since the outbreak of Coronavirus disease 2019 (COVID-19), wearing a facial mask has been a usual topic, and wearing a facial mask could result in a reduction of exposure to airborne pollutants and nasal rhinitis symptoms, with fewer allergic symptoms in the general population (22). Therefore, it could be speculated that wearing protective devices such as a facial mask could affect the epistaxis. In current study we included the epistaxis cases happened before COVID-19 period to rule out the effect of wearing protective devices on occurrence of epistaxis. Therefore, comparing the relationship between each environmental factors with epistaxis before, and after COVID-19 could make another interesting results.

This study has some limitations. First, this study was conducted using health insurance claims, and owing to the nature of the study data, there is a possibility that enrolled cases in the current study do not completely reflect all true cases of epistaxis. It has not captured the data of patients who had episodes of epistaxis but were not registered in the national portal. Therefore, the results may be underestimated or overestimated compared to the actual number of patients. Second, we only evaluated meteorological and air pollutant factors that are regularly monitored in Korea; other potential environmental risk factors that could affect the occurrence of epistaxis were not considered in this study (23). Third, we compared the association between epistaxis and environmental factors measured daily but did not assess data regarding the possible delayed effects of each environmental factor on epistaxis in each age group. Finally, the statistical analysis has been performed by stepwise regression. This was performed to manage the large number of potential predictor variables to choose the best predictor variables from the available options. Multicollinearity is a known problem in stepwise regression analysis when a number of intercorrelated predictors are entered into a regression model. Usually, the greatest limitation of this procedure would be the sample size (24). However, in this study, stepwise regression was performed in a large sample size which could have circumvented this problem. Regardless of this, the results of our study need to be interpreted with caution.

To conclude, this study has served to highlight the association between environmental factors and epistaxis, and more importantly, it has shown that the effects are more pronounced in the younger age group.

## Data availability statement

The original contributions presented in the study are included in the article/Supplementary material, further inquiries can be directed to the corresponding authors.

## Ethics statement

The studies involving human participants were reviewed and approved by the Institutional Review Board of Chung-Ang Hospital (2005-015-19314) and the review board waived the requirement for informed consent. Written informed consent from the participants' legal guardian/next of kin was not required to participate in this study in accordance with the national legislation and the institutional requirements.

## Author contributions

HM: concept and design, drafting of the manuscript, critical revision of the manuscript for important intellectual content, and supervision. E-JA: acquisition, analysis, or interpretation of data, drafting of the manuscript, critical revision of the manuscript for important intellectual content, and statistical analysis. All authors contributed to the article and approved the submitted version.

## Funding

This work was supported by the National Research Foundation of Korea (NRF) grant funded by the Korea government (MSIT) (2022R1F1A1063720 to HM).

## Conflict of interest

The authors declare that the research was conducted in the absence of any commercial or financial relationships that could be construed as a potential conflict of interest.

## Publisher's note

All claims expressed in this article are solely those of the authors and do not necessarily represent those of their affiliated organizations, or those of the publisher, the editors and the reviewers. Any product that may be evaluated in this article, or claim that may be made by its manufacturer, is not guaranteed or endorsed by the publisher.
