# Supplementary material for: Age-specific associations between environmental factors and epistaxis
Source: Front Public Health. 2022 Oct 19;10:966461. doi: 10.3389/fpubh.2022.966461 (PMC9626808; doi:10.3389/fpubh.2022.966461)
Supplement: Supplementary file 1 [file Table_1.DOCX]

Supplementary Material

| **Meteorological factors** | | | | | | | | | |
| --- | --- | --- | --- | --- | --- | --- | --- | --- | --- |
| **Month** | **Mean temperature (°C)** | **Average ground temperature (°C)** | **Lowest temperature (°C)** | **Highest temperature (°C)** | **Average wind speed (m/s)** | **Maximum wind speed (m/s)** | **Sunshine duration (h)** | **Average relative humidity (%)** | **Average atmospheric pressure (hPa)** |
| 1 | -2.09 [4.3] | -1.72 [2.5] | -5.53 [4.5] | 1.79 [4.3] | 2.40 [0.8] | 4.87 [1.3] | 5.53 [3.3] | 55.59 [13.2] | 1014.05 [4.6] |
| 2 | 0.99 [4.3] | 1.37 [2.8] | -2.70 [4.4] | 5.33 [4.6] | 2.59 [0.8] | 5.27 [1.4] | 5.87 [3.5] | 53.59 [14.3] | 1012.3 [5.2] |
| 3 | 5.81 [3.9] | 6.98 [3.6] | 1.74 [3.8] | 10.56 [4.6] | 2.88 [0.9] | 5.90 [1.5] | 6.27 [3.8] | 53.56 [14.3] | 1008.71 [5.4] |
| 4 | 12.33 [3.6] | 14.04 [3.8] | 7.95 [3.2] | 17.39 [4.4] | 2.90 [0.9] | 6.22 [1.6] | 6.36 [4.2] | 54.26 [15.7] | 1004.38 [5.2] |
| 5 | 18.37 [2.8] | 21.19 [3.7] | 13.76 [2.7] | 23.65 [3.6] | 2.61 [0.8] | 5.60 [1.4] | 7.01 [4.5] | 58.14 [15.1] | 1000.69 [4.8] |
| 6 | 22.79 [2.2] | 25.91 [3.5] | 18.90 [2.1] | 27.56 [3.2] | 2.37 [0.7] | 5.16 [1.1] | 5.98 [4.3] | 64.16 [14.0] | 997.47 [4.1] |
| 7 | 24.95 [2.1] | 26.28 [2.8] | 22.14 [1.9] | 28.48 [2.9] | 2.45 [0.9] | 5.14 [1.4] | 3.27 [3.6] | 76.32 [9.9] | 996.27 [4.2] |
| 8 | 25.89 [2.3] | 27.19 [2.9] | 22.87 [2.3] | 29.65 [2.9] | 2.37 [0.9] | 4.96 [1.5] | 4.47 [3.9] | 72.87 [9.9] | 998.56 [3.6] |
| 9 | 21.65 [2.4] | 23.25 [2.9] | 17.87 [2.6] | 26.10 [3.0] | 2.08 [0.7] | 4.48 [1.4] | 5.60 [4.1] | 65.83 [12.1] | 1003.93 [4.1] |
| 10 | 15.19 [3.5] | 15.99 [3.6] | 10.85 [3.7] | 20.28 [3.8] | 2.12 [0.7] | 4.62 [1.3] | 6.80 [3.2] | 60.29 [11.2] | 1008.87 [4.2] |
| 11 | 7.68 [4.5] | 7.13 [4.1] | 3.95 [4.6] | 11.87 [4.8] | 2.42 [0.9] | 5.01 [1.5] | 5.14 [3.4] | 60.09 [14.2] | 1011.32 [4.7] |
| 12 | -0.29 [4.7] | -0.30 [3.3] | -3.75 [4.8] | 3.58 [4.8] | 2.47 [0.8] | 4.97 [1.4] | 5.46 [3.2] | 57.16 [12.3] | 1013.33 [5.1] |
| **Air pollutants** | | | | | | | | | |
| **Month** | **PM_10_ (ppm)** | **CO (ppm)** | **O_3_ (ppm)** | **SO_2_ (ppm)** | **NO_2_ (ppm)** |  |  |  |  |
| 1 | 100.83 [48] | 1.44 [0.6] | 0.02 [0.01] | 0.011 [0.01] | 0.057 [0.02] |  |  |  |  |
| 2 | 111.62 [86] | 1.36 [0.6] | 0.03 [0.01] | 0.01 [0.005] | 0.058 [0.02] |  |  |  |  |
| 3 | 128.23 [109] | 1.05 [0.5] | 0.038 [0.01] | 0.0091 [0.005] | 0.059 [0.02] |  |  |  |  |
| 4 | 131.44 [164] | 0.95 [0.4] | 0.049 [0.02] | 0.008 [0.004] | 0.065 [0.02] |  |  |  |  |
| 5 | 108.68 [72] | 0.95 [0.3] | 0.059 [0.02] | 0.0078 [0.004] | 0.064 [0.02] |  |  |  |  |
| 6 | 85.25 [47] | 0.83 [0.3] | 0.064 [0.03] | 0.0063 [0.005] | 0.056 [0.02] |  |  |  |  |
| 7 | 70.48 [42] | 0.83 [0.4] | 0.046 [0.03] | 0.0051 [0.004] | 0.045 [0.02] |  |  |  |  |
| 8 | 58.93 [36] | 0.76 [0.3] | 0.0459 [0.03] | 0.0046 [0.002] | 0.043 [0.01] |  |  |  |  |
| 9 | 61.68 [40] | 0.82 [0.4] | 0.043 [0.02] | 0.0055 [0.002] | 0.051 [0.02] |  |  |  |  |
| 10 | 79.58 [46] | 1.12 [0.5] | 0.036 [0.01] | 0.0071 [0.003] | 0.059 [0.02] |  |  |  |  |
| 11 | 93.28 [87] | 1.29 [0.5] | 0.025 [0.01] | 0.0089 [0.004] | 0.057 [0.02] |  |  |  |  |
| 12 | 97.76 [83] | 1.37 [0.6] | 0.02 [0.01] | 0.01 [0.004] | 0.056 [0.02] |  |  |  |  |

Values are presented as mean [standard deviation].

Abbreviations: PM_10_, particulate matter with diameter ≤10 µm; CO, carbon monoxide; O_3_, ozone; SO_2_, sulfur dioxide; NO_2_, nitrogen dioxide

eTable 1. **Monthly mean values of meteorological factors and air pollutants**

|  | **Age group 0**  **(N=19,580)** | **Age group 1**  **(N=10,978)** | | **Age group 2**  **(N=13,395)** | **Age group 3**  **(N=2,675)** |  |
| --- | --- | --- | --- | --- | --- | --- |
| Age | 8.59 [4.4] | 28.24 [6.3] | | 53.56 [8.3] | 75.61 [4.6] |  |
| Sex (male/female) | 10,179/9,401 | 5,382/5,596 | | 6,986/6,409 | 1,355/1,320 |  |
| **Comorbidities** |  |  | |  |  |  |
| Chronic sinusitis | 12,172 (62.1) | | 12,172 (62.1) | 6,012 (54.7) | 6,664 (49.7) | |
| Acute sinusitis | 17,332 (88.5) | | 17,332 (88.5) | 8,219 (74.8) | 8,592 (64.1) | |
| Chronic rhinitis | 11,156 (56.9) | | 11,156 (56.9) | 6,148 (13.2) | 6,732 (50.2) | |
| Septal deviation | 1,752 (8.9) | | 1,752 (8.9) | 2,783 (25.3) | 2,453 (18.3) | |

Values are presented as mean [standard deviation] or number (percentage).

eTable 2. **The prevalence of comorbidities in each age group**
